# Modulatory Role of Hesperetin–Copper(II) on Gut Microbiota in Type 2 Diabetes Mellitus Mice

**DOI:** 10.3390/foods14132390

**Published:** 2025-07-06

**Authors:** Xi Peng, Yushi Wei, Deming Gong, Guowen Zhang

**Affiliations:** 1 State Key Laboratory of Food Science and Resources, Nanchang University, Nanchang 330047, China; pxjxja@126.com (X.P.); weiyushi99@163.com (Y.W.); dgong01@gmail.com (D.G.); 2 Department of Biological Engineering, Jiangxi Biotech Vocational College, Nanchang 330200, China

**Keywords:** hesperetin–copper(II) complex, type 2 diabetes, gut microbiota

## Abstract

Background: Exploring new strategies to improve type 2 diabetes mellitus (T2DM) is one of the frontier hotspots in the field of healthy food. Flavonoid–metal complexes have become one of the research hotspots in the field of health foods due to their unique structural and functional properties. Methods: In this study, the effect of hesperetin–copper(II) complex [Hsp–Cu(II)] on the gut microbiota of mice with T2DM was investigated by the 16S rRNA high-throughput sequencing. Results: The analyses of α and β diversity indicated that the richness and diversity of gut microbiota in the T2DM mice decreased and the community structure was significantly different from the normal mice. Hsp–Cu(II) increased the abundances of the beneficial bacteria (*Lactobacillus*, *Ligilactobacillus*, *Romboutsia*, *Faecalibaculum*, and *Dubosiella*), and decreased the amounts of the harmful bacteria (*Desulfobacterota*, *Corynebacterium*, and *Desulfovibrio*) and the ratio of *Firmicutes*/*Bacteroidetes* (from 44.5 to 5.8) in the T2DM mice, which was beneficial for regulating the composition of intestinal microbiota. The linear discriminant analysis effect size analysis showed that the intervention of Hsp–Cu(II) made the short-chain fatty acid (SCFA) producers (*o_Lachnospirales*, *f_Lachnospiraceae*, *g_Faecalibaculum*, *g_Romboutsia*, and *g_Turicibacter*) and the lactic acid bacteria producers (*f_Lactobacillaceae* and *o_Lactobacillales*) highly enriched, and the production of its metabolite SCFAs (acetic acid, propionic acid, butyric acid, and valeric acid) were increased in a dose-dependent manner, promoting the SCFA metabolism. Conclusions: Hsp–Cu(II) may improve glucose metabolic disorders and alleviate T2DM by modulating gut microbiota composition, promoting probiotics proliferation and SCFAs production, restoring intestinal barrier integrity, and suppressing local inflammation. These research findings may provide a theoretical basis for developing Hsp–Cu(II) as a new hypoglycemic nutritional supplement, and offer new ideas for the dietary food nutritional regulation to alleviate T2DM.

## 1. Introduction

Microorganisms are important for keeping human health [1]. Current research has confirmed that the gut microbiota participates in the fundamental biological processes of humans [2]. Chronic diseases, like obesity and diabetes, are related to the human microbiota [3]. At the same time, energy balance and the immune system also rely on the functions of the microbiota [4]. Moreover, the gut microbiota ferment indigestible carbohydrates, like dietary fiber, and produce important metabolites, like short-chain fatty acids (SCFAs), which are of great significance to human health [5].

It has been reported that T2DM was related to intestinal microecological imbalance, and the gut microbiota composition of patients with type 2 diabetes differs from that of healthy individuals [6,7]. In particular, there were changes in the abundances of *Firmicutes* and *Bacteroidota*, which were usually manifested as follows: the presence of more *Firmicutes*, along with a higher ratio of *Firmicutes* to *Bacteroidota* (F/B) [8], and a decrease in the *Bacteroidota* abundance. For instance, Larsen et al. [9] discovered that the number of *Firmicutes* in patients with T2DM increased, while the numbers of *Clostridium* and *Bacteroidetes* decreased. These changes in the number of bacteria may be related to abnormal blood glucose levels in the host [10]. It was also reported by Karlsson et al. [11] that a lower number of *Lactobacillus* and a higher number of *Clostridium* were found in T2DM patients compared with healthy people. The gut microbiota of 345 Chinese people was studied by Qin et al. [12], who found that the structure of the microbiota was disordered in diabetic patients, which in turn had a further impact on the occurrence and development of T2DM. The imbalance of gut microbiota has also been reported as a possible cause of insulin resistance [13]. Most dietary polyphenols reach the intestine to perform physiological functions, such as regulating the gut microbiota and improving T2DM. Estruel-Amades et al. [14] reported that hesperidin regulated the intestinal flora composition of healthy rats and increased the number of *Lactobacillus* and *Staphylococcus*. Zhang et al. [15] found that epigallocatechin gallate (EGCG) and (-)-gallocatechin gallate (GCG) significantly promoted the growth of *Bifidobacteria*, increased the ratio of *Lactobacillus* to *Enterococcus faecalis*, and inhibited the growth of *Prevotella* and *Clostridium perfringens* at the same time, thus enhancing the intestinal proboscis and improving intestinal health. The metabolite ferulic acid has been shown to reduce the abundance of *Firmicutes* and to increase the abundance of *Bacteroidetes* in the intestines of mice on a high-fat diet. This results in a decreased *Firmicutes*/*Bacteroidetes* ratio and alleviates symptoms of T2DM [16,17]. Licorice extract regulated the gut microbiota of T2DM mice by reducing the abundances of *Lachnospiraceae_NK4A136_group*, while increasing the abundances of *Akkermansia* and *Bacteroides* [18]. The polyphenol extract from *Dendrobium* ameliorated the diabetic symptoms in mice due to its ability to decrease inflammation as well as to enhance the equilibrium of the intestinal microflora [19]. The polymethoxyflavones from citrus increased the abundances of *Bacteroides ovatus*, *Bacteroides uniformis*, and *Bacteroides thetaiotaomicron*, which helped relieve T2DM [20].

SCFAs are the major products of soluble dietary fiber under the action of specific gut microbiota. It was generally believed that SCFAs were metabolic targets. SCFAs regulate energy balance, improve inflammation, protect pancreatic islet β-cells, prevent intestinal disorders, and enter the systemic circulation as signaling molecules [21,22], affecting the host metabolism. The well-defined mechanism by which SCFAs regulate T2DM is to activate the G protein-coupled receptors (GPRs) on adipocytes, intestinal immune cells, and intestinal epithelial cells to boost the body’s ability to handle glucose and the sensitivity of the hormone insulin [23]. Alternatively, they can activate GPR43 to inhibit insulin-induced Akt phosphorylation in adipose tissue, thereby improving insulin sensitivity [24]. Once the intestinal microbiota is disrupted, the reduced production of SCFAs may cause changes in the intestinal microenvironment, affecting the energy metabolism of carbohydrates and fats, which may lead to T2DM. Qin et al. [12] found that the content of microbiota-produced SCFAs was significantly decreased in T2DM and obesity. Xu et al. [25] reported that Kombucha alleviated T2DM by modulating SCFA-producing bacteria.

Hesperetin is a kind of polyphenol that is widely found in fruits and vegetables. Our previous results have indicated that Hsp–Cu(II) synthesized from hesperetin and copper(II) showed stronger inhibition activities of starch-digesting enzymes than the ligand hesperetin, and possessed a significant hypoglycemic effect in vitro [26,27]. However, there has been no research looking at the effect of Hsp–Cu(II) on the gut microbiota of mice with T2DM.

Thus, the objective of this research was to investigate the effects of Hsp–Cu(II) on the microbial flora and SCFAs of the T2DM mice to explore the possible regulatory role of Hsp–Cu(II) on intestinal disorders. The correlation between SCFAs and gut microbiota in T2DM was also studied to probe the possible hypoglycemic mechanism of Hsp–Cu(II). The findings of this study could provide useful references for developing Hsp–Cu(II) as a hypoglycemic nutritional supplement for the alleviation of T2DM.

## 2. Materials and Methods

### 2.1. Chemicals and Reagents

Hesperetin–Copper(II) was synthesized and characterized according to our previous study [28]. The analytically pure standard products of acetic acid, propionic acid, butyric acid, and valeric acid were purchased from Aladdin Biochemical Technology Co., Ltd. (Shanghai, China). The kits for D-lactic acid (A019-3-1) and diamine oxidase (DAO, A088-3-1) were bought from Nanjing Jiancheng Bioengineering Institute (Nanjing, Jiangsu, China).

### 2.2. Animals

The C57 BL/6J mice (SPF grade, 6–8 weeks, male) were bought from Xiaoshuyoutai Biotechnology Co., Ltd. [Beijing, China; License number: SCXK (Jing) 2023-0010]. The animal experiments were approved by the Ethics Committee of Laboratory Animals of Nanchang University [SYXK (Gan) 2021-0004] on 13 July 2021. After one week of adaptive feeding on a standard diet, the T2DM mouse model was established according to our previous work by feeding a high-fat diet for 8 consecutive weeks, then combined with intraperitoneal injection of streptozotocin at 50 mg/kg for 3 consecutive days [27]. The mice were randomly (random numbers were generated using the RAND function in Microsoft Excel) divided into the Diseased group (gavaged with an equal dose of 0.5% sodium carboxymethylcellulose), the Met group (gavaged with 200 mg/kg/day metformin), the LHC group (gavaged with 10 mg/kg/day Hsp–Cu(II)), the MHC group (gavaged with 25 mg/kg/day Hsp–Cu(II)), and the HHC group (gavaged with 50 mg/kg/day Hsp–Cu(II)) (*n* = 10 per group) based on glucose levels, metformin, and Hsp–Cu(II) that were dissolved in 0.5% sodium carboxymethylcellulose. The Normal group was composed of 10 normal mice (gavaged with an equal dose of 0.5% sodium carboxymethylcellulose). All the mice were housed under identical conditions (temperature: 23 ± 2 °C; humidity: 60% ± 10%; 12 h light/dark cycle). The fasting blood levels in the T2DM mice were higher than 11.1 mmol/L. After four weeks of gavage, the fasting blood levels in the Met, LHC, MHC, and HHC groups were decreased by 51.6%, 7.8%, 21.3%, and 32.3%, respectively. Then, all the mice were fasted for 12 h and sacrificed after being injected with pentobarbital sodium. The blood and colon contents were immediately collected and stored at −80 °C for testing [27].

### 2.3. Determination of Diamine Oxidase (DAO) Activity and D-Lactic Acid (D-LAC) Content

Briefly, the levels of DAO and D-LAC were determined using 20 and 60 μL of serum according to the respective instructions of the kits.

### 2.4. Detection of SCFAs in the Colon Contents

A total of 100 mg of colon contents were put into a 2 mL test tube, 1 mL of water and grinding beads were added, then grinded at 60 HZ for 5 min, ultrasonicated in an ice-water bath for 30 min and stood for 20 min, then centrifuged at 10,000× *g*, 4 °C for 10 min to collect the supernatant. The steps were repeated again, and the supernatants of the two experiments were combined and mixed well. Then, a gas chromatograph (GC-2010, Shimadzu, Tokyo, Japan) was applied to measure the contents of four SCFAs (acetic acid, propionic acid, butyric acid, and valeric acid). The chromatographic column was DB-FFAP, the column temperature was 80 °C, the inlet temperature was 250 °C, and the detector temperature was 280 °C. The detection procedure specified that the temperature increased from 40 °C to 120 °C at 40 °C/min, then increased to 230 °C at 10 °C/min, and was maintained for 3 min [29].

### 2.5. Effect of Hsp–Cu(II) on the Colon Intestinal Microbiota of the T2DM Mice

The colon contents were submitted to Novogene Technology Co. (Beijing, China) for 16S rDNA high-throughput sequencing. The total genomic DNA of the microbial community was extracted and the DNA concentration and purity were determined by agarose gel electrophoresis (Biowest, Nuaillé, Pays de la Loire, France). The PE250 sequencing was performed on a NovaSeq 6000 sequencer. The α diversity indices (Chao 1, Shannon, and Simpson) were calculated. Principal component analysis (PCA) and principal coordinates analysis (PCoA) were used to gain a clearer understanding of the similarities and differences in the microbial community structures among the different samples. The Spearman method was used to analyze the correlations between the top 20 bacterial genera in relative abundance and SCFAs [30].

### 2.6. Statistical Analysis

The results are expressed as a mean ± standard deviation (SD). Data were analyzed by one-way ANOVA followed by Tukey’s post hoc test using the statistical software SPSS Statistics 26.0 (International Business Machines Corporation, Amunk, NY, USA). A significant difference between groups was determined at *p* < 0.05.

## 3. Results

### 3.1. Effect of Hsp–Cu(II) on the Intestinal Mucosal Barrier Function of T2DM Mice

**Figure 1:**
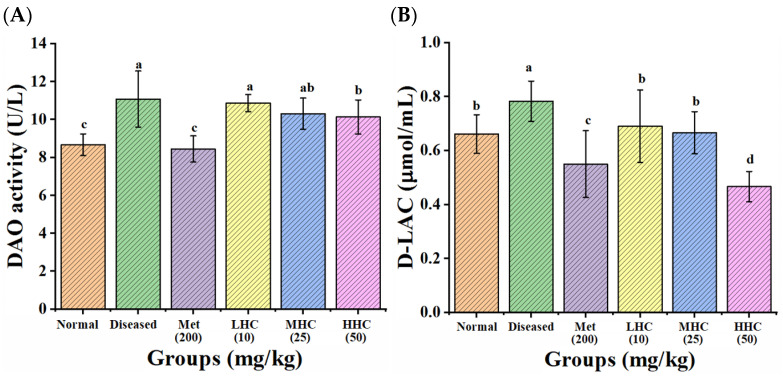
Activity of diamine oxidase (**A**) and content of D-LAC (**B**) in the serum of mice (*n* = 8). Different letters indicate a significant difference (*p* < 0.05).

Diamine oxidase (DAO) is primarily found in small intestinal mucosal epithelial cells. D-lactic acid (D-LAC) is a metabolite produced by the gut bacteria. Under normal circumstances, DAO and D-LAC cannot easily pass through the intact intestinal barrier into the bloodstream, so the serum levels of DAO and D-LAC are extremely low. When intestinal mucosal permeability is increased, DAO and D-LAC are released into the bloodstream, leading to elevated serum levels. As shown in Figure 1, the activity of DAO and the content of D-LAC in the Normal group were 8.67 ± 0.57 U/L and 0.66 ± 0.07 μmol/mL, which were significantly increased to 10.80 ± 1.48 U/L and 0.78 ± 0.07 μmol/mL in the Diseased group, respectively, suggesting that the gastrointestinal barrier of the T2DM mice was impaired. In the Met and the LHC, the MHC, and the HHC groups, the activities of DAO decreased to 8.45 ± 0.68 U/L, 10.87 ± 0.45 U/L, 10.31 ± 0.81 U/L, and 10.13 ± 0.90 U/L, respectively; while the content of D-LAC reduced to 0.55 ± 0.12, 0.69 ± 0.13, 0.66 ± 0.07, and 0.46 ± 0.05 μmol/mL, respectively, showing that Hsp–Cu(II) improved intestinal integrity and mucosal barrier function [31].

### 3.2. Effect of Hsp–Cu(II) on SCFAs in the Colon Contents of T2DM Mice

**Figure 2:**
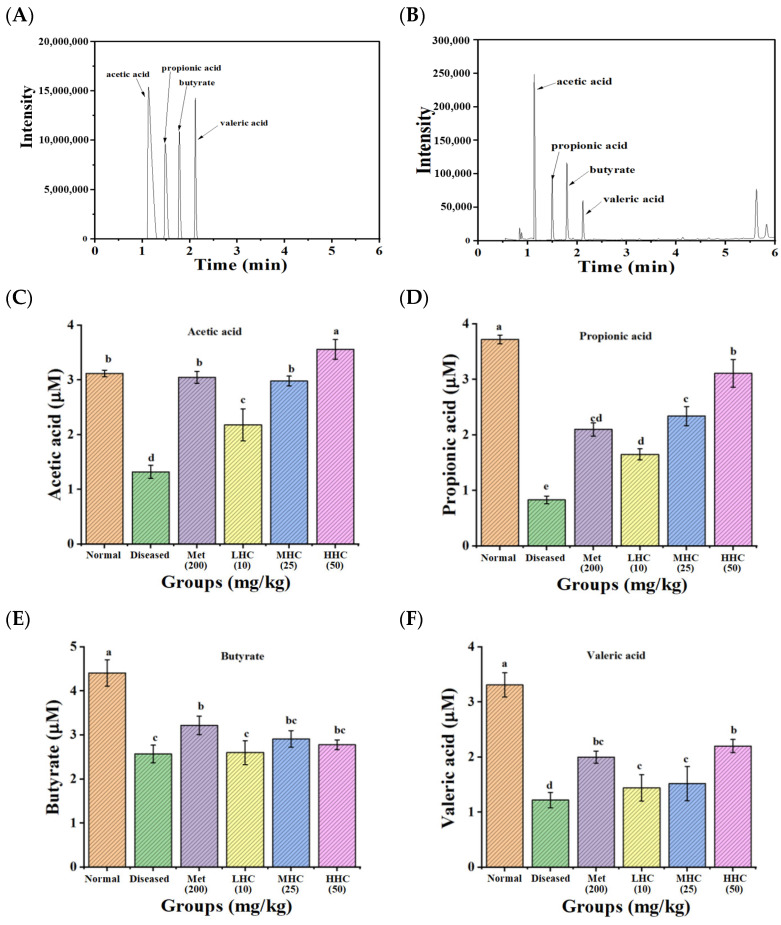
Gas chromatogram of four SCFAs of the standard (**A**) and sample (**B**). Concentration of acetic acid (**C**), propionic acid (**D**), butyric acid (**E**), and valeric acid (**F**) in the colon contents of mice (*n* = 8). Different letters indicate a significant difference (*p* < 0.05).

In Figure 2, the contents of four SCFAs (acetic acid, propionic acid, butyric acid, and valeric acid) in the Normal group were 3.12 ± 0.06, 3.72 ± 0.18, 4.41 ± 0.30, and 3.21 ± 0.22 μM, respectively, which were decreased to 1.32 ± 0.12, 0.83 ± 0.07, 2.57 ± 0.20, and 1.22 ± 0.14 μM in the Diseased group, respectively. Hsp–Cu(II) significantly increased the content of the four SCFAs in the T2DM mice. Among them, the HHC group showed the best effect on the increase of the SCFAs contents with acetic acid (3.56 ± 0.18 μM), propionic acid (3.11 ± 0.25 μM), butyric acid (2.78 ± 0.11 μM), and valeric acid (2.20 ± 0.12 μM), for which the contents of acetic acid even exceeded the Normal mice. Hsp–Cu(II) may restore the fermentative bacterial activity in the intestine, thus increasing the production of SCFAs. The promotion of intestinal the SCFA metabolism by Hsp–Cu(II) may be an important factor in its anti-diabetic effect.

### 3.3. Effect of Hsp–Cu(II) on a Diversity of Intestinal Microbiota in T2DM Mice

**Figure 3:**
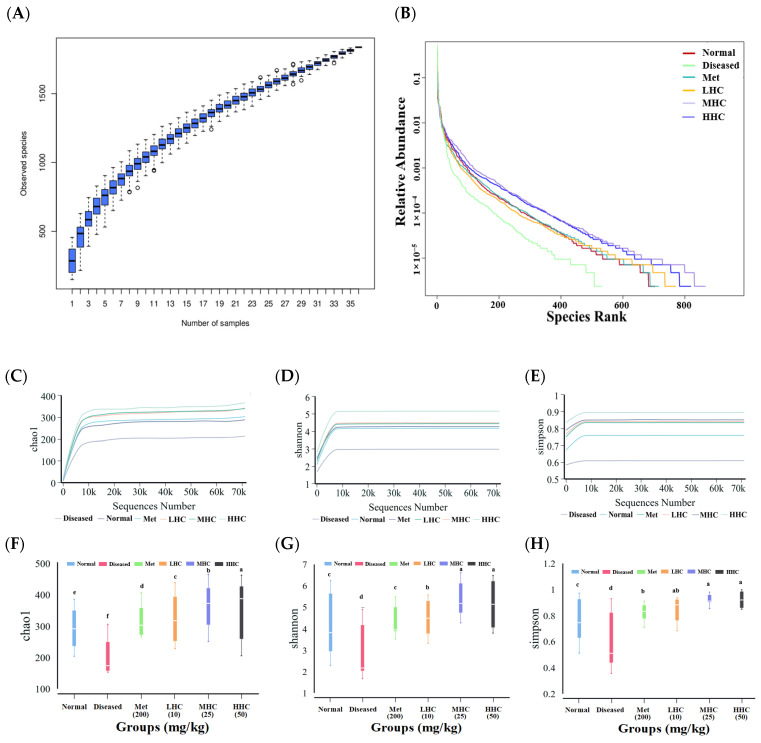
Species of accumulation curves (**A**); rank abundance curves (**B**); α diversity curves for (**C**) Chao1, (**D**) Shannon, and (**E**) Simpson; α diversity difference for (**F**) Chao1, (**G**) Shannon, and (**H**) Simpson (*n* = 6). Different letters represent a significant difference (*p* < 0.05).

A species accumulation curve analysis was conducted to explore the composition of the samples [32]. At the initial stage, the curve rose sharply as the sample size increased (Figure 3A), indicating that a wide range of species were found. After the sample size reached a certain level, the curve flattened, indicating that the number of samples was sufficient (a total of 36 samples), and the data analysis could proceed. The rank clustering curve is shown in Figure 3B. In the horizontal direction, the wider the span of the curves, the higher the richness of the species; in the vertical direction, the flatter the curves, the more even the distribution of the species [33]. The treatment of Hsp–Cu(II) increased the width of the curves in the horizontal direction, suggesting that the species abundance of intestinal microbiota in the Hsp–Cu(II) groups was higher than the Diseased group. Moreover, the curves were flatter in the vertical direction, suggesting that the species distribution was more even in the Hsp–Cu(II) groups.

The α diversity distribution of the samples can be analyzed by the Chao1, Shannon, and Simpson indices, which reflect species richness, species diversity, and evenness of distribution [32]. The three indices of each group gradually flattened with the increasing depth, indicating that the number of samples was reasonable, and the α diversity reached saturation (Figure 3C–E). In the Normal group, the Chao1 index (288.26 ± 28.55), Shannon index (3.81 ± 0.69), and Simpson index (0.75 ± 0.18) were greater than the Diseased group (204.08 ± 15.27, 2.19 ± 0.48, and 0.51 ± 0.25, respectively) (*p* < 0.05) (Table 1 and Figure 3F–H), suggesting that the α diversity of the T2DM mice was reduced. After intragastric administration of Hsp–Cu(II), the three indices were greater than the Diseased group, and the effect was most obvious in the HHC group, with its Chao1 index (359.77 ± 35.32), Shannon index (5.19 ± 0.93), and Simpson index (0.92 ± 0.04). The colonic microbiota of the T2DM mice was found to be richer and more diverse by Hsp–Cu(II).

### 3.4. β Diversity

**Figure 4:**
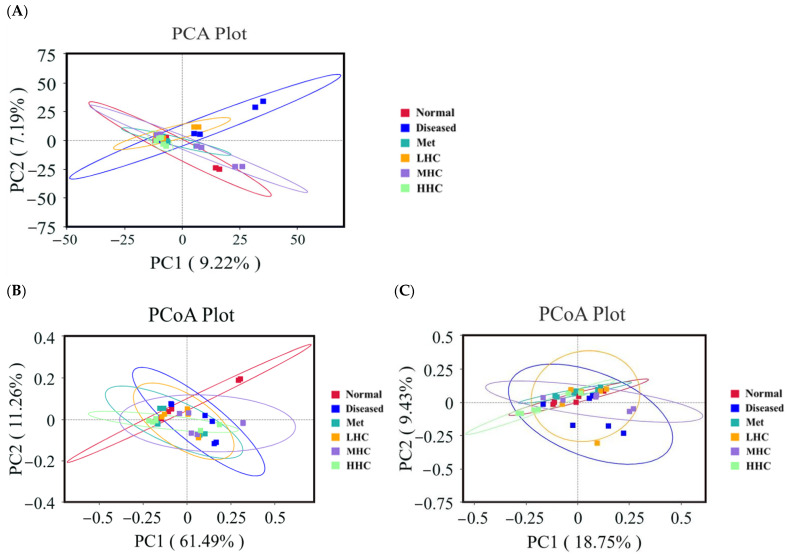
PCA analysis of all groups (**A**); PCoA analysis of all groups based on the weighted UniFrac distance (**B**); and unweighted UniFrac distance (**C**) (*n* = 6).

The number of species in a community was extremely large, and the differences in samples were often multi-dimensional, making them very difficult to compare. Therefore, the dimensionality of the multi-dimensional data was reduced using PCA [34]. If the compositions of the samples are similar, their distances in the PCA plot are close. The Normal group was mainly distributed in the second and fourth quadrants, while the Diseased group was located in the first quadrant (Figure 4A). The Normal group was separated from the Diseased group. The LHC group was located in the first and second quadrants, the MHC group was located in the second and fourth quadrants, and the HHC group was located in the second and third quadrants. These findings indicated that, after the intervention of Hsp–Cu(II), the community structure of the T2DM mice changed, but the HHC group was close to the Normal group. PCoA, the most classic unconstrained ordination analysis method [35], projects the sample distance matrix and then unfolds it in a low-dimensional space, while retaining the distance relationships of the original samples to the greatest extent. As shown in Figure 4B, all groups were analyzed using PCoA, with the weighted UniFrac distance used as the basis for the analysis. The samples for the Normal group were distributed in the second quadrant, while the distribution of the Diseased group was primarily concentrated in the first and fourth quadrants. Figure 4C shows the PCoA analysis of all groups based on the unweighted UniFrac distance. The Normal group was distributed in the second quadrant, while the Diseased group was concentrated in the first and fourth quadrants, and the samples in the Hsp–Cu(II) groups were mainly concentrated in the first and second quadrants. The samples in the Normal group were completely separated from the Diseased group. The flora structures in the diseased mice were different from the normal mice. The treatment of Hsp–Cu(II) improved the composition of the colonies in the T2DM mice.

### 3.5. Species Composition

**Figure 5:**
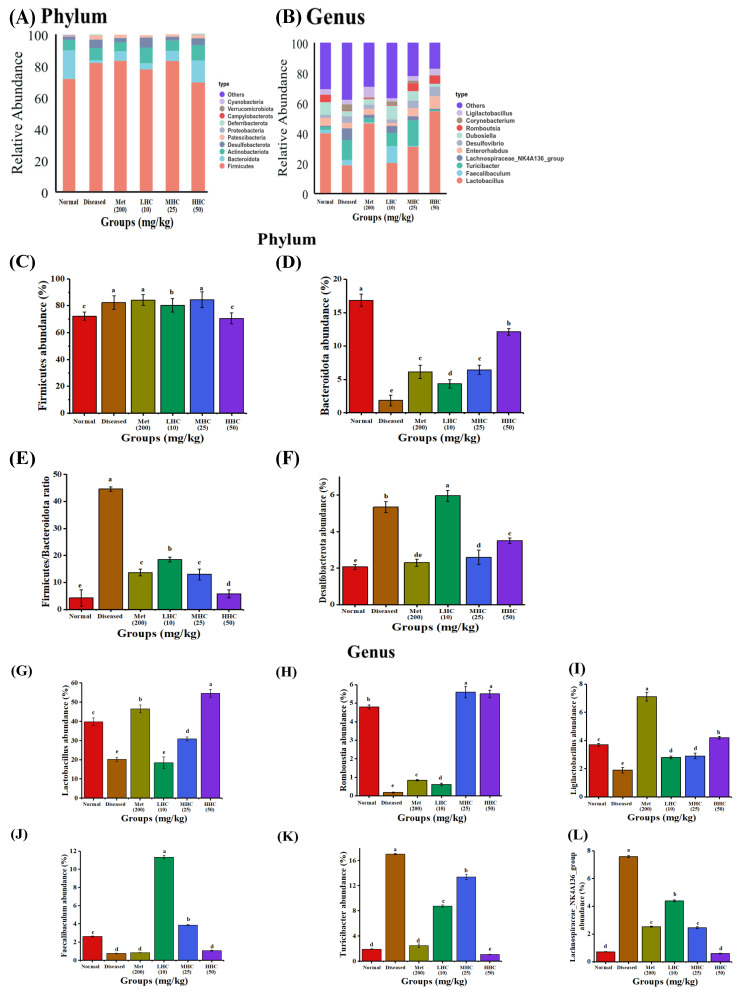

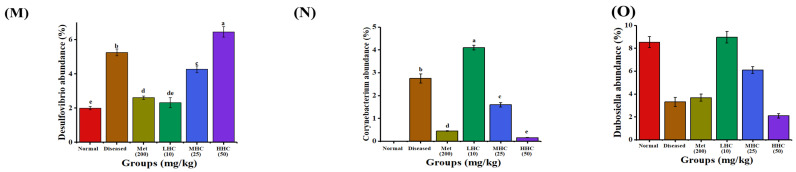
The composition of species at the phylum level (**A**) and genus-level (**B**). The relative abundance of *Firmicutes* (**C**), *Bacteroidetes* (**D**), value of F/B ratio (**E**), *Desulfobacterota* (**F**), *Lactobacillus* (**G**), *Romboutsia* (**H**), *Ligilactobacillus* (**I**), *Faecalibaculum* (**J**), *Turicibacter* (**K**), *Lachnospiraceae_NK4A136_group* (**L**), *Desulfovibrio* (**M**), *Corynebacterium* (**N**), and *Dubosiella* (**O**), (*n* = 6). Different letters indicate a significant difference (*p* < 0.05).

The colonic flora of all groups was mostly made up of *Firmicutes*, *Bacteroidota*, *Actinobacteria*, and *Desulfobacterota* (Figure 5A) at the phylum level. In the Diseased group, the abundances of *Firmicutes* (82.35%) and *Desulfobacterota* (5.34%) were higher than the Normal group (*Firmicutes*: 72.35% and *Desulfobacterota*: 2.07%), while *Bacteroidota* (1.85%) was less than the Normal group (16.38%) (Figure 5C–F). After the T2DM mice were treated with Hsp–Cu(II), the amount of *Bacteroidota* was effectively increased, especially in the HHC group, with the most significant increase to 12.15%. The abundances of Firmicutes and *Desulfobacterota* in the HHC group were reduced to 70.60% and 3.51%, respectively. *Desulfobacterota* is a harmful phylum and is toxic to the intestinal epithelium, leading to gastrointestinal diseases [36]. *Bacteroidota* is a typical beneficial phylum. A higher ratio of F/B in the intestine leads to more efficient absorption of calories from food, thus resulting in T2DM [37]. Fu brick tea ameliorated the gut microbiota and metabolic dysbiosis in mice with T2DM by reducing the F/B ratio [38]. In the present study, the F/B values of each group from low to high were as follows: Normal (4.49) < HHC (5.8) < MHC (13.03) < Met (13.7) < LHC (18.54) < Diseased (44.60). That is, the Normal group was the lowest (4.49), and the Diseased group significantly increased the F/B value (44.60). The F/B values in the Hsp–Cu(II) groups were significantly smaller than the Diseased group. Hsp–Cu(II) significantly raised the abundance of beneficial *Bacteroidota* and decreased the abundance of harmful *Desulfobacterota* and the value of the F/B ratio, which improved the intestinal colony composition of the T2DM mice.

At the genus level, the colonic flora of the groups was composed of Lactobacillus, Faecalibaculum, Turicibacter, Lachnospiraceae_NK4A136_group, Enterorhabdus, Desulfovibrio, Dubosiella, Romboutsia, Corynebacterium, and Ligilactobacillus (Figure 5B). In the Diseased group, the abundance of Turicibacter (17.05%), Lachnospiraceae_NK4A136_group (7.58%), Corynebacterium (2.75%), and Desulfovibrio (5.25%) were higher than the Normal group (Turicibacter: 1.93%; Lachnospiraceae_NK4A136_group: 0.73%; Corynebacterium: 0; and Desulfovibrio: 2.04%), while the abundances of Lactobacillus (20.31%), Romboutsia (0.18%), Ligilactobacillus (1.92%), Faecalibaculum (0.77%), and Dubosiella (3.32%) were less than the Normal group (Lactobacillus: 39.82%; Romboutsia: 4.80%; Ligilactobacillus: 3.71%; Faecalibaculum: 2.61%; Dubosiella: 8.54%) (Figure 5G–O). The treatments of the T2DM mice with Hsp–Cu(II) reversed these changes. In the HHC group, the abundances of Lactobacillus, Romboutsia, and Ligilactobacillus rose to 54.60%, 5.51%, and 4.22%, respectively; while Turicibacter, Lachnospiraceae_NK4A136_group, and Corynebacterium decreased to 1.07%, 0.61%, and 0.16%, respectively. The abundances of Faecalibaculum (11.35%) and Dubosiella (8.96%) in the LHC group showed the most increase, which exceeded the Normal group (Faecalibaculum: 2.61%; Dubosiella: 8.54%). In addition, the abundance of Desulfovibrio in the Met (2.61%), LHC (2.32%), and MHC (4.27%) groups was lower than the Diseased group (5.25%). Hsp–Cu(II) mitigated T2DM by modulating the composition of the intestinal microbiota.

### 3.6. Key Species Composition

**Figure 6:**
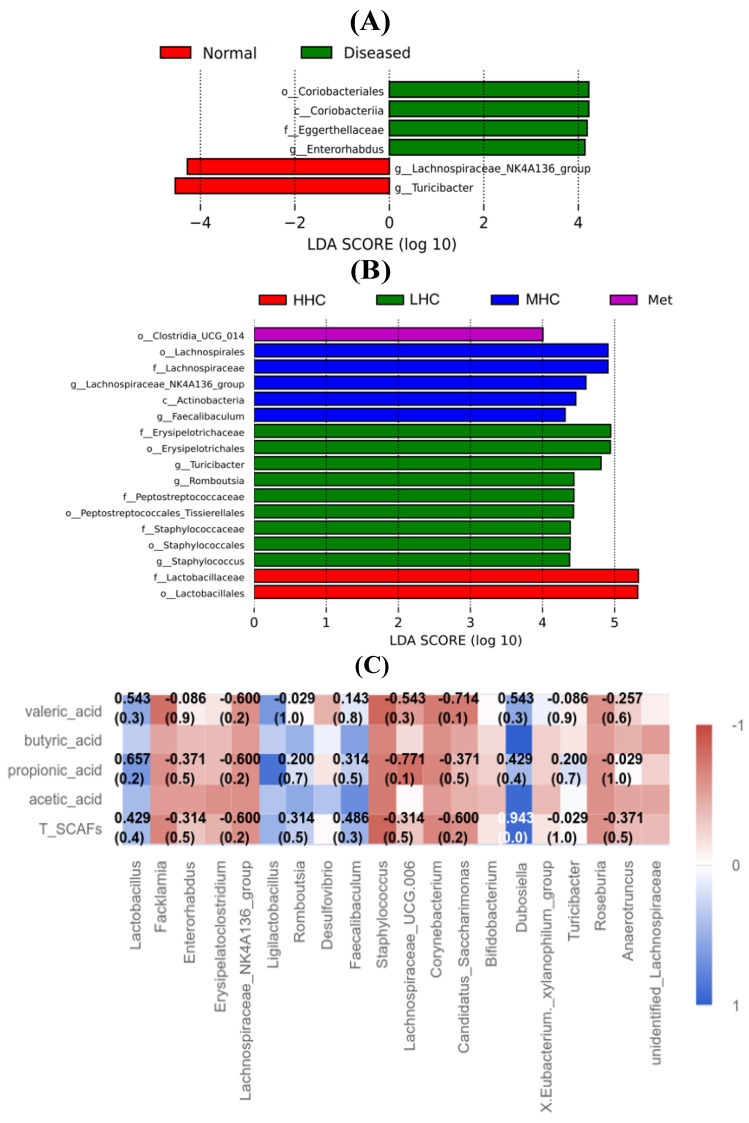
LEfSe analysis of all samples yielded information on taxonomic units with significant differences between the groups: Normal group and Diseased group (**A**); Met, LHC, MHC, and HHC groups (**B**) (*n* = 6). Heatmap of Spearman correlation between the top 20 bacteria in relative abundance and SCFAs, color changes from red (negative correlation) to blue (positive correlation) (**C**) (*n* = 6).

Linear discriminant analysis effect size (LEfSe) analysis was conducted to screen for significantly different bacteria in the different groups. The linear discriminant analysis (LDA) classification diagram and score histogram showed the dominant species in each group (Figure 6A,B). In the Normal group, *g_Turicibacter* and *g_Lachnospiraceae_NK4A136_group* were the major bacteria, while in the Diseased group, the *o_Coriobacteriales*, *c_Coriobacteriia*, *f_Eggerthellaceae*, and *g_Enterorhabdus* were the dominant bacteria. The *f_Erysipelotrichaceae*, *o_Erysipelotrichale*, *g_Turicibacter*, *g_Romboutsia*, *f_Peptostreptococcaceae*, *o_Peptostreptococcales_Tissierellales*, *f_Staphylococcaceae*, *o_Staphylococcales*, and *g_Staphylococcus* were enriched in the LHC group. The *o_Lachnospirales*, *f_Lachnospiraceae*, *g_Lachnospiraceae_NK4A136_group*, *c_Actinobacteria*, and *g_Faecalibaculum* were enriched in the MHC group, and the *f_Lactobacillaceae* and *o_Lactobacillales* were enriched in the HHC group. T2DM significantly altered the microbial community structure. After the intervention with Hsp–Cu(II), the SCFAs producers, such as *o_Lachnospirales*, *f_Lachnospiraceae*, *g_Faecalibaculum*, *g_Romboutsia*, and the lactic acid bacteria producers, like *f_Lactobacillaceae* and *o_Lactobacillales*, were highly enriched. Therefore, Hsp–Cu(II) may promote the multiplication of beneficial bacteria and intestinal health.

### 3.7. Correlation Between Intestinal Microbiota and SCFAs Production

The Spearman correlation was analyzed on the top 20 bacteria in relative abundance and levels of SCFAs. As shown in Figure 6C, *Lactobacillus* and *Ligilactobacillus* were lactic acid-producing bacteria, while *Faecalibaculum*, *Romboutsia* and *Dubosiella* were SCFA-producing bacteria, which were positively correlated with the levels of the four SCFAs. *Corynebacterium*, a harmful bacterium, was negatively correlated with the levels of the four SCFAs [39]. Another harmful bacterium, *Desulfovibrio*, was also negatively related to the contents of acetic acid and butyric acid [40]. Thus, Hsp–Cu(II) promoted the generation of SCFAs by modulating the relative abundance of beneficial bacteria (*Lactobacillus*, *Ligilactobacillus*, *Romboutsia*, *Faecalibaculum*, *Romboutsia*, and *Dubosiella*) and harmful bacteria (*Corynebacterium* and *Desulfovibrio*) to alleviate T2DM.

## 4. Discussion

Our previous study had demonstrated that Hsp–Cu(II) reduced fast blood glucose and lipid levels in the T2DM model mice by improving hepatic glucose metabolism through suppression of gluconeogenesis and promotion of glycogen synthesis [27]. However, whether this complex affected the gut microbiota in mice with T2DM remained unclear. Thus, this research aimed to elucidate the regulatory mechanisms of Hsp–Cu(II) on the gut microbiota in vivo. T2DM patients exhibit certain physiological changes, such as gut microbiota dysbiosis [6,7]. The colonic microbiota of the T2DM mice was found to be less diverse and rich than that of the normal mice in this study. Moreover, the abundances of *Firmicutes*, *Desulfobacterota*, and the value of F/B ratio in the T2DM mice were well raised, and the abundance of the *Bacteroidota* decreased. The abundance of *Turicibacter*, *Lachnospiraceae_NK4A136_group*, *Corynebacterium*, and *Desulfovibrio* increased, while *Lactobacillus*, *Romboutsia*, *Ligilactobacillus*, *Faecalibaculum*, and *Dubosiella* decreased. The results suggested the dysbiosis of the gut microbiota in the T2DM mice.

T2DM can be alleviated by modulating the diversity of gut microbiota. This has gradually become a novel approach for preventing and treating T2DM. The gut microbiota affects T2DM in multiple ways, including (1) by regulating the inflammatory response, (2) by improving intestinal permeability, (3) by producing SCFAs, and (4) by regulating the composition of bile acids [41].

T2DM could trigger a low-grade metabolic inflammatory response in the body [42]. Regulating the gut microbiota through various inflammasome components was not only a vital factor in the deterioration of fatty liver disease but an important cause affecting metabolic syndromes, such as body weight gain, imbalance of glucose homeostasis, and subclinical inflammation [43]. The gut microbiota regulated the inflammatory response by increasing the contents of pro-inflammatory factors, chemokines, and inflammatory proteins. For example, IL-10 secreted by *Lactobacillus plantarum* and *Lactobacillus casei* improved glucose metabolism [44]. The multiple lactic acid bacteria (*Lactobacillus plantarum*, *Lactobacillus* G15 and Q14) decreased the contents of IL-1β and TNF-α [19,45]. In addition, the gut microbiota also triggered an inflammatory response by activating or recognizing relevant receptors, stimulating serine (one of the insulin receptor substrates) to phosphorylate its residues, thereby reducing insulin sensitivity [46], or regulating the balance of T-cells through SCFAs [47]. As demonstrated in our previous study, the treatment of the T2DM mice with Hsp–Cu(II) resulted in a significant decrease in the levels of IL-1β, IL-6, and TNF-α [27]. In this study, Hsp–Cu(II) enriched the lactic acid bacteria (*f_Lactobacillaceae* and *o_Lactobacillales*) (Figure 6A,B) and increased the abundances of *Lactobacillus* and *Ligilactobacillus*, which are well-known probiotics that ferment sugar to produce a large amount of lactic acid [48]. Similarly, rambutan peel polyphenols increased the abundance of *Lactobacillus* to improve T2DM [49]; baicalin effectively increased the abundances of *Ligilactobacillus*, *Lactobacillus*, and *Bacteroides*, and alleviated pulmonary inflammation in rats [50]. *Corynebacterium*, a harmful bacterium, may potentially induce intestinal inflammation [40]. Chen et al. [51] reported that, after treatment with polysaccharides from *Ganoderma lucidum*, the abundance of the harmful bacterium *Corynebacterium* in the intestine of T2DM rats was decreased. *Desulfovibrio*, another harmful bacterium, absorbed sulfates and produced hydrogen sulfide, which is toxic to the intestinal epithelium, thus leading to gastrointestinal diseases [40]. Ren et al. [52] reported that the resistant starch of rice downregulated the number of *Desulfovibrio* to ameliorate T2DM. Our study showed that the abundances of *Corynebacterium* and *Desulfovibrio* in the T2DM mice were higher than those observed in the normal mice. Hsp–Cu(II) partially counteracted the increase in the number of these two bacteria in mice with T2DM. Thus, Hsp–Cu(II) improved glucose metabolism and suppressed the production of factors that cause inflammation by raising the abundance of some lactic acid bacteria and suppressing some harmful bacteria.

SCFAs were largely produced by the fermentation of non-starch carbohydrates and were either rapidly absorbed by the colonic epithelial cells or released from the intestine, which played crucial roles in regulating food and energy intake [53]. Among them, the most important were acetic acid, propionic acid, and butyric acid. Acetate was released into the peripheral tissues by affecting the secretion of intestinal hormones [54]; propionate was absorbed primarily in the liver and regulated blood glucose by activating gluconeogenesis [55]. Except for the main energy source for the colonic mucosa, butyrate also had anti-inflammatory and anti-tumor effects [55]. *Faecalibaculum* produced butyric acid that had anti-inflammatory properties and protected the digestive system from intestinal pathogens [45]. Proanthocyanidins, rutin, and polyphenol extract from *Rosa Roxburghii* fruit increased the abundance of *Faecalibaculum* to alleviate the symptoms of T2DM and regulated gut microbiota [56,57]. *Romboutsia* was also a genus of butyric acid-producing bacteria, which participated in regulating blood glucose homeostasis, and made an impact on the development of diabetes [58]. *Dubosiella* showed effects, such as regulating in vivo metabolism, improving immunity of the intestinal tract and strengthening the resistance of the body to inflammatory diseases, which had an impact on the various life activities of individuals [45]. A study has shown that *Dubosiella* was positively correlated with the level of acetate [59]. In this study, Hsp–Cu(II) enriched the SCFA producers (*o_Lachnospirales*, *f_Lachnospiraceae*, *g_Faecalibaculum*, *g_Romboutsia*, and *g_Turicibacter*) and led to a rise in the quantity of *Romboutsia*, especially in the MHC and HHC groups. The amount of *Faecalibaculum* and *Dubosiella* in the LHC group exhibited the greatest increase. However, the abundance of *Turicibacter* (producing butyric acid) in the Hsp–Cu(II) groups was lower than the Diseased group, which needed further research. Moreover, the four SCFAs were positively correlated with *Faecalibaculum*, *Romboutsia*, and *Dubosiella*. Thus, Hsp–Cu(II) may alleviate T2DM mainly by promoting the SCFAs metabolism.

In T2DM, the intestinal barrier is destroyed by the combined effects of gut microbiota dysbiosis and dietary factors [60]. High-fat and high-sugar diets not only led to T2DM but caused an imbalance in the intestinal microflora and an increase in intestinal permeability [61]. Our study demonstrated that the levels of DAO and D-LAC in the T2DM mice were 1.24 and 1.18 times greater than those of the normal mice, indicating that the gastrointestinal barrier function of the T2DM mice was impaired, and intestinal permeability increased. It was found that *Bacteroides vulgatus* and *Bacteroides dorei* were beneficial to diabetes, resulting in a decrease in intestinal permeability [62]. Cano et al. [63] also reported that *Bacteroidota* improved the metabolic function in obese mice. Butyric acid produced by *Faecalibaculum* and *Roseburia* reduced intestinal permeability through the 5-hydroxytryptamine transporter and the PPAR-γ pathways [64]. Our study showed that Hsp–Cu(II) increased the abundances of *Bacteroidota* and *Faecalibaculum* in the T2DM mice, and the levels of DAO and D-LAC in the Hsp–Cu(II) groups were lower than those in the Diseased group, indicating that Hsp–Cu(II) significantly decreased intestinal permeability and improved the intestinal mucosal barrier function to alleviate T2DM. These findings demonstrate that the hypoglycemia of Hsp–Cu(II) changed the composition and structure of gut microbiota, enriching SCFA-producing bacteria and promoting the SCFA metabolism, suppressing the production of inflammatory factors and reducing intestinal permeability.

## 5. Conclusions

The intestinal mucosal barrier function of mice with T2DM was enhanced by Hsp–Cu(II), and the levels of four SCFAs (acetic acid, propionic acid, butyric acid, and valeric acid) were increased. The colonic microbiota of the T2DM mice was richer and more diverse after treatment with Hsp–Cu(II), which led to an increased amount of *Bacteroidota*, a decreased amount in *Firmicutes* and *Desulfobacterota*, and a reduction in the value of the F/B ratio. At the same time, Hsp–Cu(II) increased the abundance of helpful bacteria (*Lactobacillus*, *Romboutsia*, *Ligilactobacillus*, *Faecalibaculum*, and *Dubosiella*) and reduced the abundance of harmful bacteria (*Corynebacterium* and *Desulfovibrio*). The *o_Coriobacteriales*, *c_Coriobacteriia*, *f_Eggerthellaceae*, and *g_Enterorhabdus* were dominated in the T2DM mice. The SCFAs-producing bacteria (*o_Lachnospirales*, *f_Lachnospiraceae*, *c_Actinobacteria*, *g_Faecalibaculum*, *g_Turicibacter*, and *g_Romboutsia*) and lactic acid bacteria (*f_Lactobacillaceae* and *o_Lactobacillales*) were highly enriched in the LHC, MHC, and HHC groups. Moreover, the abundances of *Lactobacillus*, *Ligilactobacillus*, *Romboutsia*, *Faecalibaculum*, *Dubosiella*, and *Turicibacter* were positively correlated with the levels of the four SCFAs, and the abundance of the harmful bacterium *Corynebacterium* was negatively correlated with them. Therefore, Hsp–Cu(II) improved glucose metabolic disorders and alleviated T2DM by modulating gut microbiota composition, enriching the beneficial bacteria, and promoting the production of SCFAs, reducing the amount of harmful bacteria, suppressing local inflammation, and restoring intestinal barrier integrity. This study could lay the theoretical groundwork for developing Hsp–Cu(II) as a new functional food factor with hypoglycaemic properties and food dietary nutritional supplements.

## Figures and Tables

**Table 1:** α diversity indexes of sample species (*n* = 6).

Group Names	Chao 1	Shannon	Simpson
Normal	288.26 ± 28.55 ^e^	3.81 ± 0.69 ^c^	0.75 ± 0.18 ^c^
Diseased	204.08 ± 15.27 ^f^	2.19 ± 0.48 ^d^	0.51 ± 0.25 ^d^
Met	316.68 ± 7.67 ^d^	3.94 ± 0.83 ^c^	0.82 ± 0.07 ^b^
LHC	323.33 ± 5.97 ^c^	4.48 ± 0.95 ^b^	0.88 ± 0.10 ^ab^
MHC	347.08 ± 8.27 ^b^	5.18 ± 0.79 ^a^	0.91 ± 0.06 ^a^
HHC	359.77 ± 35.32 ^a^	5.19 ± 0.93 ^a^	0.92 ± 0.04 ^a^

Note: Different letters indicated the significant difference (*p* < 0.05).

## Data Availability

The original contributions presented in the study are included in the article. Further inquiries can be directed to the corresponding author.
